# The future of DNA sequence archiving

**DOI:** 10.1186/2047-217X-1-2

**Published:** 2012-07-12

**Authors:** Guy Cochrane, Charles E Cook, Ewan Birney

**Affiliations:** 1 EMBL-Bioinformatics Institute, Wellcome Trust Genome Campus, Hinxton, CB10 1SD, United Kingdom

**Keywords:** DNA, Sequence, Archive, Compression, Storage, Image

## Abstract

Archives operating under the International Nucleotide Sequence Database Collaboration currently preserve all submitted sequences equally, but rapid increases in the rate of global sequence production will soon require differentiated treatment of DNA sequences submitted for archiving. Here, we propose a graded system in which the ease of reproduction of a sequencing-based experiment and the relative availability of a sample for resequencing define the level of lossy compression applied to stored data.

## Background

The vast majority of living organisms utilise nucleic acid as their primary store of genetic information. The technology to sequence DNA routinely was developed in the 1970s, but advances over time have since reduced cost and increased output. As the cost of sequencing has fallen, the number of species for which partial or complete genetic information has been derived has risen at a corresponding pace; starting with the first complete sequence of the Phi X 174 virus [1] in 1977, the first complete bacterial genome, that of *Haemophilus influenzae*[2], in 1995 and followed by genomes of hundreds of other organisms, including eukaryotes such as humans. Currently the International Nucleotide Sequence Database Collaboration (INSDC, http://www.insdc.org/) databases hold complete genomes from 5,682 organisms and sequence from almost 700,000 organisms.

The intracellular enzymatic processes that manipulate DNA molecules are highly formulaic: this has allowed the development of sophisticated, flexible, and ever cheaper laboratory techniques in which DNA and RNA can be cut, ligated, interconverted and replicated *in vitro*. Coupled with the decreasing cost of sequencing, DNA has become a convenient readout for a variety of molecular biology assays. This started with the development of EST and cDNA technologies, was followed by high-throughput genome sequencing and then progressed through routine large-scale transcriptome sequencing, and finally to yet more intensive processes such as RNA-seq, Chip-seq and DNaseI-seq. We have even witnessed the development of DNA sequencing-based methods with no direct biological role, such as the mathematical exploration of a combinatoric space and the development of unique synthetic tags for property tracking.

DNA sequences determined for research purposes have been routinely archived since 1982, when the EMBL Data Library was founded. This was closely followed by the formation of GenBank first at the US Department of Energy and then transferred to NIH, and in 1987 by the DNA Databank of Japan. These three centres joined to form a tripartite collaboration, the INSDC, to archive and provide access to all DNA sequences generated by publicly funded research [3]. This data archiving project has gone through many changes in its 30-year history, responding both to advances in sequencing technology and to changes in the use of DNA sequence information. The archived DNA sequences form one of the bedrocks of modern biological science, and are the basis of our understanding of the molecular processes of all life. The common sharing of this information worldwide has been repeatedly acknowledged as enabling new, unforeseen science, as well as providing open data for the entire life science community to build upon.

The most recent technological advances in DNA sequencing pose some new challenges. These advances are often labelled as “next generation” sequencing, although this term is likely to become less useful as the technology is continually evolving. The routine, low cost generation of large data volumes produces challenges in laboratory logistics and management as well as in data analysis. In addition, large data volumes create issues in archival storage, which so far have been mitigated by the development of specialised archival resources such as the INSDC Sequence Read Archive [4] and algorithmic developments, such as compression [5]. An additional property of advances in sequencing technology is that at the current rates of change, DNA sequencing costs will fall so low as to become negligible for some applications. This will allow a far greater range of scientific experiments to be carried out, but will also allow whimsical or nonsensical uses of DNA sequencing, and will generate additional pressure on storage resources.

This parallels recent developments in imaging technology, which also have very low costs of data acquisition for a given technological investment. Scientists who use imaging technologies must decide which images, and at what level of data loss in terms of compression, to archive. There is currently no analogue for images of the globally accessible DNA sequence archive, although there are some well developed plans to create partial or federated image archives 6. Arguably one aspect which makes the creation of centralised image archives more complex is that the open-ended nature of image acquisition allows a potentially unlimited data flow into such an archive. As the cost of sequencing decreases the already existing global DNA sequence archives also face potentially unlimited data inflows.

Currently the INSDC archives accept all DNA sequences that submitting scientists present as being relevant and publicly available; often this is due to the need for deposition mandated by journal policies, but many sequences are deposited without associated publications and, frequently, with no direct plan for publication. A concern brought by this open-ended acceptance policy is that all DNA sequences are treated identically in terms of archiving method and hence incur equal archiving costs. With the development of lossy compression based systems, sequence archives now have the ability to make quite dramatic changes in the on-disk footprint of submitted data at different levels of acceptable data loss [5]. Again, the analogy with image-based techniques is relevant, with perhaps only the most valuable images stored in a completely lossless manner even locally, with more routine storage at variable levels under lossy compression formats.

In this perspective piece, we explore the utility of different schemes for data reduction for a DNA sequence archive. The most extreme scheme uses complete data loss (e.g., storing only an analytical result deduced from the DNA sequence), but more relevant to the current situation is the large range of possible compression strategies, which offer up to 1,000-fold increased compression for DNA sequence with aggressive data loss strategies. We set out a framework in which to make data loss decisions, and explore the consequences of these decisions.

## Main text

### *Framework for archiving*

Simple and utilitarian thinking must be applied to archiving DNA sequence data. Archiving of experimental data is valid when the cost of archiving for a given experiment is lower than the cost of reproducing the experiment at some point in the future. Importantly, this balance considers only the costs of archiving and experimental reproduction; the cost of generating the data to be archived is not a factor in this decision, although, of course, knowing this cost will be helpful in the estimation of experimental reproduction.

The archiving costs can be split into two components. Firstly, there is the infrastructure cost of running the archive and providing useful access at any desired point in the future. Secondly, there is the marginal cost of storing the data items on disk. This is where compression strategies can help to reduce the cost. As there has been a consistent drop in disk storage costs over the last two decades, meaning that future disk costs per storage unit (i.e. megabyte) are a small proportion of current costs, currently one can compute the overall disk cost for a presumed “infinite” lifetime of storage; this is around 1.3x the cost of disk on day 1, with 77% of this cost falling within the first 3 years given current disk doubling rates.

The experimental data cost is more variable, but broadly one can imagine three different components. Firstly there is the acquisition or development of the samples needed for the experiment. Second, there is the experiment itself in terms of reagents and technician and other scientific time. Finally, there is the data acquisition process, which should include the marginal cost of technicians and machine cost amortisation as well as the more obvious reagent costs. It is the last cost which one can be relatively confident will continue to fall during the coming years.

In the past, the relatively high cost of sequencing (and hence high cost of reproduction of an experiment) meant that in addition to archiving being more easily justified, the other two components of the experimental cost were normally also reasonably high. In other words, whimsical DNA sequencing experiments were rarely undertaken, making it even easier to justify blanket archiving of DNA sequences with no discrimination in data compression rates for different experiments. For the rare cases in which it was perhaps inappropriate, the cost of understanding, capturing and implementing a differentiated policy on archiving was higher than any potential gain in efficiency for the archive. It is this assumption that no longer applies, leading to a differentiated view of DNA sequence archiving needs.

A third cost, that of dissemination of raw data, is revealed as a result of these changes to the DNA sequencing landscape. Traditionally, when all DNA sequence data were blanket archived, INSDC archives provided the function of dissemination of all data. This function is not trivial as it requires curation, administration of accession namespace, user support and global presentation and includes components that are inherently difficult to systematise fully and are hence costly in staff time. Since this function is implicit in running an archive in which data are made available to consumers, there was no additional visible cost for this function. However, as we move to a differentiated archiving strategy, for those data sets that will be archived in highly compressed form where dissemination requires one-off delivery of uncompressed data to consumers, an additional cost for dissemination will arise as part of the experimental data costs.

### Classification of experiments in terms of replacement cost

To achieve the goal of a classification of experiments in terms of replacement cost, it would be tempting to use monetary approaches. For example, the monetary level of the grant award from which the experiment has been supported could be used in replacement experiment valuation. This, however, will grossly underestimate some cases, in particular those which have time specific or longitudinal aspects, and grossly overestimate others, where the DNA sequencing was more for verification or was a small component of the overall experiment output. The time dependent aspects of reproducibility can be particularly important. This applies, for example, to environmental sequencing studies in which one common approach is to maintain longitudinal records for a given sampling site. While one could return in the future to the site to re-sample, one can never return to a date that has already passed and can never recreate the opportunity to integrate sequence information with contemporary contextual data, such as climatic and ecological data, for the site. (An example of this is the analysis of patterns in microbial diversity at the Western English Channel L4 site [7]).

There are similar issues in imaging. For example, satellite recording of earth images is routine, but this does not render the archive of such images from the 1960s worthless since they are time- as well as location-specific. Every experiment is of course formally a one-off event, and thus never completely reproducible, but there are variables in which the investigator (and more relevantly, a future investigator) is interested that contribute to analysis and those that are not of interest and whose fluctuation is treated by the analytical method as noise.

The other aspect is whether the DNA molecules or some derived library have been stored physically and are available for re-sequencing. From one perspective, DNA molecules offer a compact storage mechanism for sequence information and, often, stored DNA samples contain substantial regions that are yet to be sequenced. However, it is important to realise that during sequencing the DNA molecule is physically consumed and will ultimately be expended. While methods exist for replicating the molecule (such as amplification, cloning, re-synthesis, etc.), they typically provide imperfect replicates, are costly, and are not appropriate for all DNA sample types and experimental designs. The concept of physical storage of DNA molecules as an appropriate archival format seems more relevant to the original investigator as a solution for management in local sequencing projects rather than as a global archiving strategy. This is in part because sharing data by shipping DNA molecules for resequencing elsewhere is expensive and, critically, has rising costs (in line with transport fuel costs) as compared to the falling costs of data storage and transmission. Further, this is because the world lacks a viable physical, legal and economic infrastructure for globally coordinated storage and exchange of DNA samples as compared to the sophisticated data sharing infrastructure already offered by the internet.

Considering these two components we propose a two-axis classification of experiments, with axes:

1. The replacement cost of the experiment in an appropriate manner to gain equivalent scientific information

2. The presence of a large excess of DNA in a robust physical archive

These two axes would then form a grid, “archival worth”, on which data compression decisions could be made. The second axis is easier to define conceptually, although the terms “large excess” and “robust archive” will no doubt need discussion. The second axis might, for example, be defined as the presence of “greater than 10 mg of DNA in accessible form in an archive system which expects to store and ensure routine retrieval for at least 10 years” or “the ready and routine availability for at least 10 years of a precursor resource (such as a sample or a culture) from which greater than 10 mg of DNA can be extracted routinely and simply”. For the following discussion we will say that a sample is “physically archived/archivable” if these criteria are met, and use the term “PA”. All other samples we will call “physically unique” and use the term “PU”.

The first axis is more challenging to define, and our proposed classification is shown in Table 1. We have found it useful to consider analogous image based techniques for each class to help explore the consequences of archiving in this complementary space. We would be interested in opinions about this classification.

**Table 1:** Relative cost of regenerating sequences for different classes of experiments

**Class**	**Description**	**Example for DNA sequencing**	**Example for Imaging**
**1**	Historical sampling of environment or time point-specific elements	Environmental genomics studies with a longitudinal component; Pathogen sequencing from epidemics	Earth imaging; environmental imaging for longitudinal studies
**2**	Very rare objects	Ancient DNA specimens; forensic samples	Fossils; rare meteorites
**3**	Longitudinal studies which could in theory be rerun in the future but have a > 10 year horizon to recreate	RNA-seq and DNA-seq from a prospective cohort; environmental sequencing of a specific field trial/intervention in an environment	MRI scans from a prospective cohort; cell imaging from a cohort
**4**	Samples acquired from patients or animals with a high individual acquisition cost, but a conceptually continuous generation	Cancer DNA sequencing	Histology samples from Cancer
**5**	A complex experiment with > 6 month resource development	RNA-seq on a specifically created mouse gene knockout (mouse colonies stored)	Cell imaging on a specific RNAi library
**6**	A routine experiment with < 6 month resource development	RNA-seq of a standard cell line	Routine imaging of Drosophila embryos
**7**	Verification experiment as a component in an overall flow	Resequencing of insert vector	Imaging of cell lines to determine confluence levels

Relative costs decrease from class 1 through class 7.

We might then take the following classes, and apply a suggested “data compression factor”. We define this as the factor by which one should aim to compress; e.g., 2 would mean two-fold compressed, and 1 would mean uncompressed. Table 2 shows a straw man proposal of compression ratios for implementation in the near future.

**Table 2:** One possible set of DNA sequence data compression factors for the various experimental classes

**Class**	**Description**	**Rate for Physically Unique samples**	**Rate for Physically Archived/archivable Samples**
**1**	Historical sampling of environment or time specific elements	1.0	1.0
**2**	Very rare objects	1.0	1.0
**3**	Longitudinal studies which could in theory be rerun in the future but have a > 10 year horizon to recreate	1.0	2.0
**4**	Samples acquired from patients or animals with a high individual acquisition cost, but a conceptually continuous generation	1.0	10.0
**5**	A complex experiment with > 6 month resource development	10.0	100.0
**6**	A routine experiment with < 6 month resource development	20.0	200.0
**7**	Verification experiment as a component in an overall flow	1000.0	∞ (Infinite compression of data indicates no data archiving; it may, however, be useful simply to record that the experiment was carried out.)

Compression is higher for data that are easy or inexpensive to reproduce, and lower for data derived from unique or irreproducible samples.

The compression ratios here are provided to stimulate debate but relate to initial trials with compression schemes. It seems that with two-fold data compression there is little change in usability of the data for analysis, and theoretical arguments that this level of data loss is within the range of error provided by sequencing machines are strong. For higher ratios, 10-fold to 100-fold compression is achievable by aggressive treatment of stored sequence quality information with lossless sequence storage, meaning that the principle aspects of the experiment on, say, variations on a reference sequence can be executed. For compression factors greater than 100, it is likely that one would require lossy behaviour on the actual sequence, i.e. error-correction of likely sequencing errors to provide a more compressible dataset.

## Discussion

In this perspective piece we intend both to provide a framework in which to think about future DNA sequence archiving and to provide an initial opinion with concrete examples to encourage appropriate debate in the community. We believe that a broad range of scientists, funding agencies and policy makers should be interested in both topics and we welcome commentary, response or even, perhaps, cautious agreement.

A recognised value of archiving experimental data is the opportunity to support alternative analysis and metaanalysis (referred to below as ‘secondary analysis’) of the data for purposes not originally intended by the submitting scientist. Indeed, this approach has yielded useful serendipitous outputs, including an assembled genome sequence from a *Wolbachia* species discovered as contaminant sequence in *Drosophila* sequencing data, and the calling of polymorphisms in the mouse genome from archived Celera traces [8,9]. It is a feature of our proposed scheme, in which the sample is placed at the centre of the decision regarding the appropriate level of compression for a derived dataset, that the nature of the submitter’s intended analysis of sequence (referred to below as the ‘original analysis’) is not a factor in the choice of compression level. As such, any compression applied to a dataset will constrain *secondary analysis* no more than it constrains *original analysis* and the cost of regenerating the sequence necessary for a *secondary analysis* will be minimised. Given that we must accept the necessity of moving to lossy compression for archival data and the constraints on reuse that this necessarily brings, discovery of emergent properties of existing datasets, and of existing datasets in new combinations, will remain viable.

By considering the costs of DNA sequence archiving as having at least some variable component we can provide a nuanced view of archiving, and thus remain compatible with the two “common sense” positions of “one should not throw away unique, irreplaceable data” and “one should not archive verification experiments that only confirm some finding in a paper”. At the extremes this is already occurring in that for the most precious samples there is tendency to store very raw data (e.g., the Solexa images for ancient DNA samples) and there is not the expectation by authors or manuscript reviewers to, say, submit the sequence of “sequence verification” on cloning vectors. However, this framework and proposal spans these two extreme positions, and provides a rationale for the different archiving behaviours at these opposite ends of the spectrum.

We note that the biological imaging field does not have a centralised archiving system analogous to those for DNA sequences. There are probably many reasons why this is the case, but it is likely the marginal cost of data acquisition was always low enough to make the decision to archive less obvious for many data items. This made the overall landscape far more complex for image archives. It is worth noting that federation of the archive does not change the specific cost benefit analysis of archiving a particular experiment, but instead perhaps more easily connects the originating scientist to the cost of archiving. The downside of federation is that it is hard to guarantee access, in particular as individuals move between institutions, and there is duplication of infrastructure costs. The federate/centralise dichotomy is therefore independent of this debate, and has been commented on in other contexts [10]. Despite these differences, acquisition of DNA data increasingly resembles that of image data, and there has already been useful transfer of ideas (such as, for example, on compression standards and their implementation as specific metadata) in both directions which should deepen in the future.

## Authors’ contributions

All authors contributed to the assessment of past and present sequencing activity. EB and GC contributed predictions of future trends and developed the differentiated archiving concept. All authors contributed to the drafting of the manuscript.
